# Family circumstance, sedentary behaviour and physical activity in adolescents living in England: Project STIL

**DOI:** 10.1186/1479-5868-6-33

**Published:** 2009-06-11

**Authors:** Trish Gorely, Andrew J Atkin, Stuart JH Biddle, Simon J Marshall

**Affiliations:** 1 School of Sport and Exercise Sciences, Loughborough University, Loughborough, UK; 2 School of Exercise & Nutritional Sciences Faculty, San Diego State University, San Diego, California, USA

## Abstract

**Background:**

Identification of non-modifiable correlates of physical activity and sedentary behaviour in youth contributes to the development of effective targeted intervention strategies. The purpose of this research was to examine the relationships between family circumstances (e.g. socio-economic status, single vs. dual parent household, presence/absence of siblings) and leisure-time physical activity and sedentary behaviours in adolescents.

**Methods:**

A total of 1171 adolescents (40% male; mean age 14.8 years) completed ecological momentary assessment diaries every 15 minutes for 3 weekdays outside of school hours and 1 weekend day. Analysed behaviours were sports/exercise, active travel, TV viewing, computer use, sedentary socialising (hanging-out, using the telephone, sitting and talking) and total sedentary behaviour. Linear regression was employed to estimate levels of association between individual family circumstance variables and each behaviour.

**Results:**

Compared to girls from higher socioeconomic status (SES) groups, girls from low SES groups reported higher weekend TV viewing and higher weekday total sedentary behaviour. For boys, single parent status was associated with greater total sedentary behaviour compared to those from dual parent households. Boys and girls from low socio-economic neighbourhoods reported lower participation in sports/exercise compared to those living in higher socio-economic neighbourhoods.

**Conclusion:**

Associations were not consistent across behaviours or between genders. Overall, findings indicate that boys from single parent households and girls from low socio-economic families may be at increased risk of high sedentary behaviour. Those living in low socioeconomic neighbourhoods may be at increased risk of reduced participation in sports and exercise.

## Introduction

Regular physical activity is associated with small, but significant, improvements in the physical and psychological well being of young people, yet approximately 30% of boys and 40% of girls in the United Kingdom fail to meet current physical activity recommendations [1,2]. Insufficient physical activity in youth is a key public health issue, partly because patterns of behaviour established during childhood may persist into adulthood [3,4]. Moreover, the increasing prevalence of overweight and obesity in young people has been attributed, in part, to reduced physical activity and increased involvement in sedentary behaviours, such as television (TV) viewing [5]. Estimates suggest that almost a third of young people in developed countries watch more than 4 hours of TV a day [6] double the maximum dose recommended by The American Academy of Pediatrics [7]. Prolonged periods of sitting have been associated with several metabolic risk factors, independent of participation in physical activity, suggesting that the health protective effects of physical activity may be negated by prolonged bouts of sedentary behaviour [8,9]. Improved understanding of the determinants of physical activity and sedentary behaviour in youth contributes to the development of behaviour change interventions [10].

The study of sedentary behaviour as a distinct concept, rather than the mere absence of activity, has been advocated [11]. The most prevalent sedentary behaviour in youth is TV viewing and research has examined its correlates and associations with health outcomes [12,13]. However, it is unlikely that youth sedentariness can accurately be represented by one behaviour [14]. Sedentary behaviours, such as playing computer games, using the telephone, and using the internet for homework or social networking, are increasingly accessible to young people, and it is unlikely that different sedentary behaviours affect physical activity and health outcomes uniformly. For example, in a cross-sectional sample of Chinese adolescents computer non-users tended to have a more sedentary lifestyle as compared to computer users, but these relationships were stronger in males [15]. Methodologies that can capture the multiple sedentary and active behaviours that adolescents engage in are required.

The influence of family on physical activity in youth is well established [16]. Evidence suggests that family characteristics may also be associated with young people's sedentary behaviour, though most studies looking at specific sedentary behaviours have focused only on TV viewing [17-19]. Understanding how non-modifiable characteristics of the family, such as composition (single vs. dual parent, presence or absence of siblings) and socio-economic status (SES), influence health behaviour allows for targeting of 'at-risk' groups and enables researchers to tailor their interventions appropriately [10]. Few studies have explored associations between family structure, physical activity and TV viewing in adolescents. One study reported higher levels of activity amongst adolescents from single parent families [20]; but another found no association [21]. There is consistent evidence that young people from single parent families watch more TV than those from two parent families, but associations with other sedentary behaviours have not been studied [12]. In adolescents sibling physical activity has been identified as a consistent positive correlate of adolescents physical activity [16]. The relationship between TV viewing and siblings viewing levels is unclear. For example, a recent study found that adolescents with siblings were more likely to watch >2 hours of television per day [18] but a previous review had concluded that TV viewing was unrelated to being an only child [12].

Studies of the relationship between adolescent physical activity and SES have largely indicated a positive association, though findings of negative and no association are present in the literature and gender may moderate the relationship [16,22,23]. Review evidence indicates that SES, measured by parent education or income, is consistently and inversely related to TV viewing in young people [12]. However, associations with other markers of SES (father's occupation; maternal employment) were inconsistent [12]. Research exploring the influence of SES on other sedentary behaviours is lacking.

Existing evidence on associations between physical activity, sedentary behaviour and family circumstance in adolescents is inconclusive. Few studies have explored the influence of parent or sibling status on physical activity and fewer still have examined associations with sedentary behaviours other than TV viewing. A more complete understanding of how these factors influence health behaviours in adolescents may contribute to the development of interventions to increase physical activity and reduce sedentary behaviour. Therefore, the aim of the present study is to examine the relationships between family circumstance and leisure time physical activity and sedentary behaviours (assessed by Ecological Momentary Assessment (EMA)) in adolescents. EMA reduces known sources of bias inherent in other retrospective self report measures [24,25]. The physical activity behaviours examined are active travel and sports/exercise participation. The sedentary behaviours examined are television viewing, computer time (computer game playing + non-homework computer use), sedentary socialising behaviours (hanging out + sitting and talking + phone) and total sedentary time. Family circumstance variables were single or dual parent status, presence/absence of siblings, family position (youngest, middle, oldest), mother and father occupation, and highest parent occupation.

## Method

### Sampling design

Data were from a larger study of adolescent lifestyles (Project STIL – *Sedentary Teenagers and Inactive Lifestyles*) within the United Kingdom. Sampling took place between 2000 and 2002 across 15 regions stratified across England, Northern Ireland, Scotland, and Wales. Schools were randomly sampled from the largest Local Education Authority in each region, stratified by the ratio of government funded ("secondary") schools to fee-paying ("independent") schools in that Local Education Authority. To control for seasonal variation in behaviour as far as possible, sampling occurred at all schools in two waves (wave 1 predominantly March to May and wave 2 September to November) 6 months apart. Sampling procedures were designed to ensure that separate students were sampled in each wave. To further increase sample size, an additional sample was recruited 6 months after wave two using the same procedures. At each school, a study coordinator randomly sampled one class from each of three year groups: year 9 (13–14 years old), year 10 (14–15 years old), and year 11(15–16 years old). All students in the selected class were invited to participate in the study. All study procedures were approved by the Ethical Advisory Committee of the first author's institution and were in accordance with the ethical guidelines of the British Psychological Society. Informed consent was obtained from all parents/guardians and participants.

### Participants

The sample for the current study is 1171 (boys n = 477, girls n = 694) adolescents from England who returned completed diaries. The mean age of participants was 14.8 years (SD = 0.86). The sample was predominantly white-European (85.7%) broadly reflecting the racial/ethnic make-up of this school-aged population in England [26]. The proportion of participants classified as low, moderate or high SES, using the area-level index of multiple deprivation score, was 19.2%, 35.6%, and 45.2% respectively, indicating a higher than average socio-economic profile. Only participants from England were included in the current analysis as comparable postcode derived area level SES data were not available for participants from the other UK countries.

It was not possible to compute an overall response rate for the study because of missing data in the logs completed by school staff that were used to track the number of diaries distributed at each school. However, 5400 surveys were sent to 45 schools as part of Project STIL. If all surveys were distributed at all schools, this represents a response rate of 29.7%. However, this is likely to be a considerable underestimate of the true response rate because an excess of diaries were sent to each school to allow for differing class size, loss of diaries, etc., and many schools returned unused diaries or distributed them only to a single year group. As an indication of the potential scope of response rate underestimation, at the 13 schools who returned completed log books indicating the number of diaries actually handed out we calculated the response rate to be 50.2%. The terms of our ethical clearance did not allow us to collect any information about those who were eligible to participate but chose not to, hence it is unknown whether non-participants differed in anyway from participants.

### Instrumentation

The principal data collection instrument was a pencil and paper self-report diary of "free-time" that participants completed outside of school hours. Because the focus of our study involved behaviours that could be regarded as 'volitional', behaviours in school were not assessed. The self-report diary is based on principles of ecological momentary assessment and has been described in Gorely et al. 2007 [27]. The first part of the diary involved background questions about variables at the child, family, and environmental level that have been hypothesized to correlate with sedentary behaviour and physical activity. In this paper we draw on the following child and family level variables: index of multiple deprivation (IMD; this is a measure of compound social and material deprivation, calculated from a variety of data including income, employment, health, education, and housing. It is based on the postcode of the participants home, and thus represents an area level approximation of socio-economic status); who they live with (reclassified as dual or single parent household); mother's and father's occupation; highest parent occupation, number of brothers; number of sisters (reclassified as brother yes/no, sister yes/no, siblings yes/no); and age of any siblings (used to calculate family position – youngest, middle, oldest child). Part one was answered once at the start of data collection. The second part was for recording the behaviours, locations, and social contexts that the young people engaged in each day. Participants completed Part 2 of the diary for four randomly assigned days (three weekdays and one weekend day). At 15-minute intervals, participants self-reported (free-response) their main behaviour and also responded to two closed-response items, "Where are you?" (LOCATION) and "Who's with you?" (WHO). Only the behaviour data is used in the current paper. For each weekday, 44 time-samples were obtained (one every 15 minutes from 07.00 h to 08.45 h and from 15.00 h to 23.45 h). For the weekend day, 68 time-samples were obtained (one every 15 minutes from 07.00 h to 23.45 h).

To assess the reliability of the ecological momentary assessment method, participants responded to a five-point categorical item estimating the average time lag between each interval prompt and actual diary entry (5, 15, 30, 60 or >60 minutes). Only 11% of respondents reported completing each diary entry within 5 minutes of the specified interval. Fifteen percent reported completing the diary usually within 15 minutes, 17% within 30 minutes, 17% within 1 hour and 40% usually greater than 1 hour. This suggests that most participants relied on some degree of retrospective recall for recording their behaviour and the context in which it occurred but the duration of recall and subsequent effects of memory distortion are likely to be minimized using this method relative to other forms of recall self-reports[25].

### Data analysis

The behaviours were first coded into 23 categories derived inductively from our own focus group research about how English youth spend their free time and described in Gorely et al. (2007) [27]. To estimate the time spent in each behaviour category, the interval-level data were aggregated for each individual (separately by weekday and weekend day) by multiplying the daily frequency of the event by 15 (1 interval = 15 minutes). This makes an assumption that each episode of behaviour occurred for the entire 15 minutes of the sampling period. Although this may not always be true, underestimation and overestimation errors are assumed to cancel out in interval-contingent sampling schedules and, when aggregated across the day or class, yield valid estimates of duration [28].

As sedentary behaviours and physical activity differ by gender [14,29,30] analyses were conducted separately for boys and girls. Weekday and weekend day data were also analysed separately because of the greater discretionary time at weekends, which may influence behaviour choice [31,32]. All statistical analyses were carried out using Stata 8.0 (Stata Corporation, College Station, TX). To account for cluster-based sampling, Stata *survey *commands were employed. A significant difference (non-equivalence) between girls and boys for time in each behaviour was determined by non-overlapping 95% confidence intervals. Associations between TV viewing, computer time, sedentary socialising behaviours, total sedentary time, sport and exercise and active travel were estimated by Pearson's correlation. Linear regression was employed to estimate the levels of association between individual family circumstance variables and each behaviour. Within each regression analysis the Wald test was used to test the joint null hypothesis for multi-category predictor variables, producing a single p-value.

## Results

### Physical activity and sedentary behaviour

Time (min.d^-1^) in selected behaviours for weekdays and weekends are presented in Table 1. Briefly, boys reported significantly more time in sports/exercise than girls on weekdays (31 vs. 18 min.d^-1^) and weekend days (80 vs. 37 min.d^-1^). Girls reported significantly more active travel than boys at weekends only (16 vs. 11 min.d^-1^). TV viewing and computer use was significantly greater in boys than girls both during the week (TV 127 vs. 102 min.d^-1^; computer 42 vs 15 min.d^-1^) and at weekends (TV 198 vs. 154 min.d^-1^; computer 84 vs. 22 min.d^-1^). Girls spent significantly more time in social-sedentary behaviours than boys on weekdays (61 vs. 38 min.d^-1^) and weekends (161 vs. 99 min.d^-1^). Weekday and weekend total sedentary time was significantly greater in boys than girls (weekdays 207 vs 178 min.d^-1^; weekends 381 vs. 337 min.d^-1^).

**Table 1:** Time (min d^-1^) in selected behaviours for weekdays and weekends

	**Weekday**		**Weekend**	
	
	**Boys**	**Girls**	**Boys**	**Girl**
	
	Mean (95% C.I.)	Mean (95% C.I.)	Mean (95% C.I.)	Mean (95% C.I.)
**Sport and exercise**	30.9 (26.9, 34.9)^†^	18.1 (15.9, 20.3)	79.9 (70.0, 89.8)^‡^	37.2 (32.0, 42.4)

**Active travel**	21.4 (19.4, 23.4)	22.7 (21.1, 24.4)	10.5 (7.9, 13.1)^‡^	16.4 (13.9, 19.0)

**TV viewing**	127.0 (120.5, 133.5)^†^	102.3 (97.9, 106.7)	197.6 (184.9, 210.2)^‡^	153.6 (144.4, 162.7)

**Computer use**	41.9 (37.5, 46.2)^†^	14.5 (12.8, 16.3)	84.0 (73.5, 94.6)^‡^	22.1 (18.3, 26.0)

**Social-sedentary^#^**	37.7 (33.0, 42.4)^†^	61.3 (57.1, 65.4)	99.4 (87.2, 111.6)^‡^	160.9 (150.9, 170.9)

Total sedentary*	206.6 (198.5, 214.6)^†^	178.1 (172.3, 183.9)	381.0 (364.5, 397.5)^‡^	336.6 (324.6, 348.7)

* Total sedentary behaviour = TV viewing + computer use + social sedentary behaviour.

^# ^Social-sedentary behaviours = sitting and talking + talking on the telephone + shopping and hanging-out

^† ^Significant weekday difference by gender (p < .05)

^‡ ^Significant weekend difference by gender (p < .05)

### Relationships between family circumstance and sedentary behaviour

#### Total Sedentary Time

In boys, there were no significant associations between total sedentary time and any of the proxy measures of socio-economic status (see additional file 1: Table S2 Relationship between individual family circumstance variables and minutes per day in total sedentary behaviour adjusted for age and season). Among girls, parent occupation was associated with weekday total sedentary behaviour, such that girls with parents in less skilled occupations reported higher levels than the other two groups (p = .02). Girls with one or more sisters had higher levels of total sedentary time (p = .02) on weekdays. For boys, living in a single parent household was associated with greater total sedentary time on weekdays (p = .02) and weekends (p = .00). Boys with one or more brothers had lower levels of total sedentary time on weekends (p = .05).

#### Television Viewing

There were no associations for boys or girls between SES measures and TV viewing on weekdays (see additional file 2: Table S3 Relationship between individual family circumstance variables and minutes per day TV viewing adjusted for age and season). Weekend TV viewing was associated with parent occupation (p = .02) and mother occupation (p = .04) amongst girls, with lower SES or less skilled maternal employment being associated with higher viewing levels. Boys in the middle group for parent occupation (admin/skilled) reported higher weekend TV viewing (p = .00) than boys in the other two groups, and boys from single parent households reported higher levels of weekend TV viewing (p = .05).

#### Computer Use

For girls, the only variable associated with computer use was mother's occupation (see additional file 3: Table S4 Relationship between individual family circumstance variables and minutes per day using a computer adjusted for age and season). Girls with mothers in less skilled work reported lower levels of computer use at weekends, compared to the other two groups (p = .00). Parent occupation was associated with boys' computer use at weekends, with less skilled parental employment associated with lower levels of computer use (p = .04). Family position was associated with weekday computer use in boys only, with middle siblings reporting lower levels (p = .02). Boys in single parent households reported higher computer use on weekdays (p = .01).

#### Social Sedentary Behaviour

Boys' social sedentary behaviours were not associated with family circumstance on weekdays (see additional file 4: Table S5 Relationship between individual family circumstance variables and minutes per day in social sedentary behaviours adjusted for age and season). Boys whose father was in less skilled employment reported the highest levels of social sedentary behaviours at the weekend (p = .04). Less skilled parent occupation was associated with low levels of social-sedentary behaviour in girls at the weekend (p = .04), whilst less skilled maternal occupation was associated with the highest levels of social sedentary behaviour in girls on weekdays (p = .04). The only family structure variable associated with social-sedentary behaviour was for boys, with those in single parent households reporting higher levels at weekends (p = .04).

### Relationships between family circumstance and physical activity

#### Sports and Exercise

Neighbourhood SES was associated with sports/exercise for boys (p = 0.05) and girls (p = .01) on weekdays, and at weekends for girls only (p = .00) (see additional file 5: Table S6 Relationship between individual family circumstance variables and minutes per day in sports and exercise adjusted for age and season). In each case, low SES groups reported lower levels of activity. There were no associations between family structure and sports/exercise in girls. Boys with a sister (one or more) indicated lower levels of sports/exercise at the weekend (p = 0.02).

#### Active travel

Active travel was not associated with any of the family circumstance variables in girls (see additional file 6: Table S7 Relationship between individual family circumstance variables and minutes per day in active travel adjusted for age and season). For boys, maternal occupation was associated with active travel on weekends (p = .00) and weekdays (p = .00), and parent occupation associated with active travel at weekends only (p = .00). Employment in less skilled work was associated with lower levels of active transport in each case. On weekdays boys who were the youngest sibling in the family (p = .04) and on weekends boys who were an only child (p = .03) reported higher levels of active transport.

## Discussion

The present study examined the influence of family circumstance on the physical activity and sedentary behaviour of adolescents living in England. Associations were not consistent across behaviours or between genders, and relationships for variables with more than two categories were not necessarily linear. Overall, higher levels of sedentary behaviour were associated with living in a single parent household in boys and lower SES in girls. Living in a low SES neighbourhood was associated with reduced participation in sports/exercise in boys and girls, and low individual level SES was associated with lower levels of active travel in boys only. Family structure was not associated with physical activity in girls, and relationships were inconsistent in boys.

Total sedentary behaviour was greater in boys from single versus dual parent households on weekdays and at weekends. Associations with individual sedentary behaviours (e.g. TV viewing, computer use) or groups of sedentary behaviour (e.g. sedentary socialising) were consistently in this direction, though not all attained significance. Associations between single parent status and television viewing have been reported previously in boys and girls [12,19], and girls only [17]. No association between single parent status and sedentary behaviour was found for girls in the present study. Previous research exploring associations between single parent status and other sedentary behaviours, such as computer use or sedentary socialising, are not available. Adolescent boys from single parent households may be at increased risk of high levels of sedentary behaviour. Interventions that target this population group with tailored messages to reduce sedentary behaviour are recommended.

Girls from low SES backgrounds reported higher levels of sedentary behaviour than those from mid or high SES groups. Results are broadly consistent with those reported in the literature. However, previous studies have examined the influence of SES on TV viewing only [12], or employed other proxy measures for SES, such as parent education [19], therefore direct comparisons with existing evidence is difficult. Due to the composition of the study sample, the lowest SES groups often comprised relatively few participants compared with the 'mid' and 'high' SES groups, thus associations should be interpreted with caution. However, findings indicate that girls from low SES backgrounds may be at risk of high levels of sedentary behaviour, indicating a need for research to investigate sedentary behaviours more specifically in this demographic. Positioning this work alongside investigations of other health behaviours showing a similar inverse relationship with SES in children and young people in the UK (e.g., cigarette smoking, fat and fibre consumption) will help build a more complete picture of how SES influences lifestyle and subsequent health risk [33].

In this sample, sedentary socialising contributed markedly to total sedentary behaviour amongst girls, particularly at the weekend. This is consistent with findings within the larger dataset [27] and in Scottish youth [34] and reinforces the call for methodologies that capture multiple sedentary behaviours, especially in girls, because single behaviours, such as TV viewing, may not provide an accurate estimate of broader sedentary behaviour patterns.

Results indicated that living in a low SES neighbourhood was associated with reduced participation in sports/exercise in boys and girls, but individual levels of SES were not associated with participation. Many previous studies have examined the influence of SES on physical activity in young people, but no consistent pattern of association is apparent [33]. This is most likely due to the variety of objective and self-report methods used to assess activity and the application of different SES measures across studies [35]. In addition, SES may be differentially associated with different types of physical activity, based on factors such as cost, accessibility, and socio-cultural preferences. In the present study, we examined the influence of SES on two distinct domains of physical activity, namely sports/exercise and active travel. Participation in sports/exercise was consistently lower for boys and girls from low SES neighbourhoods, as determined by the IMD 'area-level' measure. Findings support previous research indicating that neighbourhood characteristics play a key role in determining youth activity patterns [36]. No associations were found between neighbourhood SES and active travel for boys or girls. There was some evidence to suggest that parent occupation (mother's occupation in particular) may be associated with active travel in boys. Lower levels of active travel for boys whose parents were employed in 'less skilled' work suggests that family level characteristics related to parent occupation may influence participation in active travel. However, this finding should be treated with caution and requires further investigation due to the relatively low number of participants in this category.

A key strength of this study was the use of EMA to assess multiple sedentary behaviours, enabling a more complete examination of associations between sedentary behaviour and family circumstance than has been reported previously. Although EMA offers benefits in terms of its ability to capture a range of behaviours, it remains a self-report approach with the potential for socially desirable responses. In addition, it is time consuming and places a higher burden on respondents compared with retrospective recalls. Individuals with psychological or behavioural problems, or with low literacy, may be less able to understand diary instructions or comply with the recording schedule [37] potentially impacting the generalisability of results. Intensity of behaviours was not assessed, as the focus of the study was the frequency and duration of behaviour, and this is acknowledged as a limitation when discussing physical activity outcomes. Notwithstanding these limitations of EMA, the greater ecological validity inherent within this approach and the richness of the data obtained are strengths of the current research that can not be obtained through most other methods. Although data were gathered from a large sample of adolescents in England, it may not be appropriate to generalise findings to other countries. Findings are based on cross-sectional data, thus causality cannot be inferred. Where family circumstance categories contain few participants, for example 'less skilled' parental occupation, associations should be interpreted with caution.

## Conclusion

To reduce health inequalities, interventions to increase physical activity and decrease sedentary behaviour should be tailored to the needs of specific risk groups. The present study examined associations between family circumstance, physical activity and sedentary behaviour in adolescents. Findings indicate that boys from single-parent families and girls from low SES groups may be at risk of high levels of sedentary behaviour and lower participation in physical activity, suggesting that interventions may need to target these specific groups. Future research that draws participants from a broad demographic, in terms of SES and family structure, may provide further insight into the influence of family circumstance on these key health related behaviours.

## Competing interests

The authors declare that they have no competing interests.

## Authors' contributions

TG participated in the design of the study, conducted the analysis and contributed to the interpretation of the data and the preparation of the manuscript. AA contributed to data interpretation and drafted the manuscript. SJHB and SJM participated in the design of the study and provided critical comment on the manuscript.

## Supplementary Material

Additional file 1 **Table S2. Relationship between individual family circumstance variables and minutes per day in total sedentary behaviour adjusted for age and season**. Results table describing the relationship between family circumstance variables and minutes per day in total sedentary behaviour. Click here for file

Additional file 2 **Table S3. Relationship between individual family circumstance variables and minutes per day TV viewing adjusted for age and season**. Results table describing the relationship between family circumstance variables and minutes per day of TV viewing. Click here for file

Additional file 3 **Table S4. Relationship between individual family circumstance variables and minutes per day using a computer adjusted for age and season**. Results table describing the relationship between family circumstance variables and minutes per day of computer use. Click here for file

Additional file 4 **Table S5. Relationship between individual family circumstance variables and minutes per day in social sedentary behaviours adjusted for age and season**. Results table describing the relationship between family circumstance variables and minutes per day in social sedentary behaviours. Click here for file

Additional file 5 **Table S6. Relationship between individual family circumstance variables and minutes per day in sports and exercise adjusted for age and season**. Results table describing the relationship between family circumstance variables and minutes per day in sports and exercise. Click here for file

Additional file 6 **Table S7. Relationship between individual family circumstance variables and minutes per day in active travel adjusted for age and season**. Results table describing the relationship between family circumstance variables and minutes per day in active travel. Click here for file
