# Supplementary material for: Family circumstance, sedentary behaviour and physical activity in adolescents living in England: Project STIL
Source: Int J Behav Nutr Phys Act. 2009 Jun 11;6:33. doi: 10.1186/1479-5868-6-33 (PMC2702353; doi:10.1186/1479-5868-6-33)
Supplement: Additional file 3 — Table S4. Relationship between individual family circumstance variables and minutes per day using a computer adjusted for age and season. Results table describing the relationship between family circumstance variables and minutes per day of computer use. [file 1479-5868-6-33-S3.doc]

**Table S4. Relationship between individual family circumstance variables** and minutes per day using a computer adjusted for age and season

| **Family Circumstance Variable** | **Boys** | | | | | | | | **Girls** | | | | | | | |
| --- | --- | --- | --- | --- | --- | --- | --- | --- | --- | --- | --- | --- | --- | --- | --- | --- |
|  | **Weekday** | | | | **Weekend** | | | | **Weekday** | | | | **Weekend** | | | |
|  | n | mean | β | p-value | n | mean | β | p-value | n | mean | β | p-value | n | mean | β | p-value |
| **Neighbourhood SES**  Low*  Medium  High | 70  163  169 | 56.3  40.2  40.0 | --  -3.9  -1.7 | .87 | 66  149  160 | 108.0  79.6  82.0 | --  -22.2  -18.7 | .12 | 123  196  287 | 15.6  13.3  15.3 | --  -2.5  -2.0 | .87 | 114  180  270 | 27.6  20.3  23.7 | --  -8.2  -2.6 | .21 |
| **Parent Occupation**  Senior position*  Admin/skilled  Less skilled | 308  106  9 | 42.8  37.6  51.9 | --  -3.5  3.7 | .88 | 285  99  8 | 81.5  83.0  43.1 | --  -7.2  -54.9 | .04 | 441  157  20 | 13.8  16.2  20.8 | --  3.4  12.0 | .25 | 411  147  16 | 21.6  23.7  32.8 | --  -1.9  -6.5 | .86 |
| **Mother Occupation**  Senior position*  Admin/skilled  Less skilled | 177  177  18 | 44.7  41.1  36.0 | --  -0.7  -10.0 | .46 | 162  167  17 | 87.6  79.7  75.9 | --  -21.3  -20.0 | .68 | 262  266  24 | 13.9  14.0  20.6 | --  2.5  10.0 | .28 | 242  251  20 | 19.8  22.4  9.8 | --  -1.2  -15.2 | .00 |
| **Father Occupation**  Senior position*  Admin/skilled  Less skilled | 250  104  28 | 43.7  34.7  41.6 | --  -7.3  -6.7 | .37 | 232  99  25 | 81.4  72.3  85.2 | --  18.4  -43.5 | .48 | 329  140  47 | 15.5  13.5  15.9 | --  -1.4  -0.9 | .43 | 309  126  42 | 24.2  23.8  16.1 | --  -2.1  -13.2 | .67 |
| **Parents at home**  Dual*  Single | 411  55 | 40.3  56.1 | --  16.5 | .01 | 386  45 | 81.3  98.3 | --  3.0 | .90 | 581  88 | 14.9  13.0 | --  -1.4 | .67 | 538  82 | 22.8  17.2 | --  -1.9 | .72 |
| **Siblings**  None*  One or more | 58  411 | 50.2  40.8 | --  -3.8 | .71 | 52  381 | 94.6  83.7 | --  -14.5 | .35 | 97  585 | 11.9  15.0 | --  -1.4 | .66 | 92  538 | 20.7  22.6 | --  5.1 | .26 |
| **Brothers**  None*  One or more | 200  269 | 45.6  39.2 | --  -6.0 | .35 | 186  247 | 89.0  82.0 | --  -12.4 | .44 | 307  375 | 13.8  15.3 | --  0.9 | .76 | 288  342 | 20.6  23.8 | --  5.5 | .12 |
| **Sisters**  None*  One or more | 213  256 | 43.8  40.4 | --  -5.5 | .37 | 198  235 | 85.7  84.4 | --  -0.3 | .98 | 335  347 | 13.4  15.7 | --  .68 | .76 | 313  317 | 22.8  21.9 | --  0.4 | .91 |
| **Family position**  Youngest*  Middle  Oldest | 121  62  210 | 41.5  35.7  43.2 | --  -9.3  14.8 | .02 | 113  57  195 | 80.2  72.6  91.0 | --  -5.9  22.4 | .44 | 181  103  288 | 14.7  16.9  14.8 | --  -.58  -1.96 | .76 | 167  91  266 | 21.4  19.1  25.4 | --  -5.8  1.4 | .40 |

* Referent category
