# Supplementary material for: Family circumstance, sedentary behaviour and physical activity in adolescents living in England: Project STIL
Source: Int J Behav Nutr Phys Act. 2009 Jun 11;6:33. doi: 10.1186/1479-5868-6-33 (PMC2702353; doi:10.1186/1479-5868-6-33)
Supplement: Additional file 6 — Table S7. Relationship between individual family circumstance variables and minutes per day in active travel adjusted for age and season. Results table describing the relationship between family circumstance variables and minutes per day in active travel. [file 1479-5868-6-33-S6.doc]

**Table S7. Relationship between individual family circumstance variables** and minutes per day in active travel adjusted for age and season

| **Family Circumstance Variable** | **Boys** | | | | | | | | **Girls** | | | | | | | |
| --- | --- | --- | --- | --- | --- | --- | --- | --- | --- | --- | --- | --- | --- | --- | --- | --- |
|  | **Weekday** | | | | **Weekend** | | | | **Weekday** | | | | **Weekend** | | | |
|  | n | mean | β | p-value | n | mean | β | p-value | n | mean | β | p-value | n | mean | β | p-value |
| **Neighbourhood SES**  Low*  Medium  High | 70  163  169 | 26.6  22.8  17.0 | --  734  -2.1 | .17 | 66  149  160 | 14.5  9.1  10.5 | --  -11.2  -8.5 | .19 | 123  196  287 | 24.5  24.0  21.6 | --  0.9  -1.2 | .69 | 114  180  270 | 21.8  17.8  12.3 | --  -4.3  -8.8 | .10 |
| **Parent Occupation**  Senior position*  Admin/skilled  Less skilled | 308  106  9 | 19.8  25.0  15.0 | --  5.8  -5.8 | .07 | 285  99  8 | 8.9  15.2  0 | --  4.5  -9.9 | .00 | 441  157  20 | 21.5  25.4  32.3 | --  4.0  2.3 | .52 | 411  147  16 | 15.8  18.3  13.1 | --  3.4  -6.5 | .14 |
| **Mother Occupation**  Senior position*  Admin/skilled  Less skilled | 177  177  18 | 20.0  23.1  11.4 | --  2.8  -12.1 | .00 | 162  167  17 | 8.1  10.1  5.3 | --  0.2  -8.7 | .00 | 262  266  24 | 22.0  23.0  27.9 | --  2.7  2.9 | .81 | 242  251  20 | 17.7  15.4  18.8 | --  0.8  -2.7 | .63 |
| **Father Occupation**  Senior position*  Admin/skilled  Less skilled | 250  104  28 | 18.9  22.8  30.5 | --  8.6  11.5 | .09 | 232  99  25 | 9.1  12.4  9.0 | --  -0.0  6.7 | .99 | 329  140  47 | 20.6  24.8  23.2 | --  3.4  1.1 | .37 | 309  126  42 | 13.9  16.0  12.9 | --  4.2  0.7 | .41 |
| **Parents at home**  Dual*  Single | 411  55 | 20.3  30.2 | --  1.7 | .75 | 386  45 | 10.1  13.7 | --  2.9 | .48 | 581  88 | 22.7  23.0 | --  -1.0 | .81 | 538  82 | 16.4  15.0 | --  -1.2 | .78 |
| **Siblings**  None*  One or more | 58  411 | 25.4  20.8 | --  -4.3 | .58 | 52  381 | 11.0  10.6 | --  6.5 | .03 | 97  585 | 22.1  22.9 | --  0.1 | .98 | 92  538 | 15.2  16.4 | --  -2.5 | .52 |
| **Brothers**  None*  One or more | 200  269 | 23.6  19.7 | --  -6.0 | .19 | 186  247 | 10.4  10.8 | --  0.9 | .83 | 307  375 | 22.5  23.0 | --  1.0 | .81 | 288  342 | 18.5  14.3 | --  -3.6 | .32 |
| **Sisters**  None*  One or more | 213  256 | 21.4  21.3 | --  -0.7 | .84 | 198  235 | 10.2  11.0 | --  4.4 | .36 | 335  347 | 23.1  22.4 | --  -1.2 | .58 | 313  317 | 16.0  16.5 | --  0.7 | .81 |
| **Family position**  Youngest*  Middle  Oldest | 121  62  210 | 24.7  16.7  19.9 | --  -11.9  -6.3 | .04 | 113  57  195 | 9.8  8.7  11.9 | --  7.4  5.3 | .44 | 181  103  288 | 22.7  21.9  23.5 | --  2.1  1.9 | .81 | 167  91  266 | 17.5  19.8  14.5 | --  8.1  -4.9 | .11 |

*Referent category
